# Study on a Novel Fault Damage Degree Identification Method Using High-Order Differential Mathematical Morphology Gradient Spectrum Entropy

**DOI:** 10.3390/e20090682

**Published:** 2018-09-07

**Authors:** Huimin Zhao, Rui Yao, Ling Xu, Yu Yuan, Guangyu Li, Wu Deng

**Affiliations:** 1 Software Institute, Dalian Jiaotong University, Dalian 116028, China; 2 Chuzhou Technical Supervision and Testing Center, Chuzhou 239000, China; 3 Traction Power State Key Laboratory, Southwest Jiaotong University, Chengdu 610031, China; 4 Guangxi Key Lab of Multi-Source Information Mining & Security, Guangxi Normal University, Guilin 541004, China; 5 Guangxi Key Laboratory of Hybrid Computation and IC Design Analysis, Guangxi University for Nationalities, Nanning 530006, China; 6 Liaoning Key Laboratory of Welding and Reliability of Rail Transportation Equipment, Dalian Jiaotong University, Dalian 116028, China

**Keywords:** high-order differential mathematical morphology entropy, fault damage degree, quantitative identification, discrimination, rolling bearing

## Abstract

A damage degree identification method based on high-order difference mathematical morphology gradient spectrum entropy (HMGSEDI) is proposed in this paper to solve the problem that fault signal of rolling bearings are weak and difficult to be quantitatively measured. In the HMGSEDI method, on the basis of mathematical morphology gradient spectrum and spectrum entropy, the changing scale influence of structure elements to damage degree identification is thoroughly analyzed to determine its optimal scale range. The high-order difference mathematical morphology gradient spectrum entropy is then defined in order to quantitatively describe the fault damage degree of bearing. The discrimination concept of fault damage degree is defined to quantitatively describe the difference between the high-order differential mathematical entropy and the general mathematical morphology entropy in order to propose a fault damage degree identification method. The vibration signal of motors under no-load and load states are used to testify the effectiveness of the proposed HMGSEDI method. The experiment shows that high-order differential mathematical morphology entropy can more effectively identify the fault damage degree of bearings and the identification accuracy of fault damage degree can be greatly improved. Therefore, the HMGSEDI method is an effective quantitative fault damage degree identification method, and provides a new way to identify fault damage degree and fault prediction of rotating machinery.

## 1. Introduction

Rolling bearing is one of the most important components of rotating machinery [1]. It shows efficacy, even in in prolonged unsuitable conditions. Fatigue spalling, pitting corrosion, and severe plastic deformation often occur in rolling bearing due to the combined effects of load, transmission, and impact [2]. At the same time, this wear causes machine breakage, outage, and other accidents. The operation of the rolling bearing directly determines whether the machine is safe, efficient, and reliable. Therefore, it is necessary to monitor the operation state, and analyze and diagnose the faults of rolling bearings.

In recent years, a variety of fault diagnosis methods have been effectively proposed to detect the types and degrees of damage to motor bearings in order to keep the machinery performing at its best and avoid abnormal event progression [3,4,5,6]. These diagnosis methods mainly include fault mechanism analysis, fault feature extraction, and fault damage degree identification using appropriate signal analysis methods. Most of these methods are qualitative analysis methods that determine whether a fault exists, and its type. There is little research on quantitative fault diagnosis, which determines the degree of fault damage and remaining life. Faults development evolves from a tiny undoing to a severe one. Therefore, quantitative fault diagnosis is an effective method to describe the fault evolution process. The existing quantitative fault diagnosis methods are finite element model method, least square method, modal expansion method, harmonic theory, information entropy and support vector machines, among others [7,8,9,10].

In recent years, the quantitative analysis of fault damage degree and fault diagnosis have attracted a wide range of researchers. Jalan et al. [11] proposed a fault diagnosis method based on a model for rotor system misalignment and mass unbalance, which used the residual generation technique and vibration signal to obtain fault state feature and fault location. Sudhakar et al. [12] used the equal force and vibration minimization method to diagnose the position and severity of unbalanced fault of the rotor system in order to solve the less vibration measurement value problem under multi-fault parameters. Lal et al. [13] developed an identification algorithm in order to estimate parameters of multiple faults in a turbine-generator system model based on forced response information. The algorithm was tested against measurement noise and found to be robust. Cui et al. [14] established a nonlinear vibration model for fault severity assessment of rolling element bearings, and proposed a quantitative fault diagnosis method. For difficult diagnostic problems focused on bearing damage size, Zhao et al. [15] proposed a quantitative diagnosis method based on empirical mode decomposition (EMD) and approximate entropy for the fault severity of rolling bearing. Ju et al. [16] proposed a quantitative diagnosis method of bearing faults based on the support vector regression method. Mathematical morphology is a nonlinear signal processing tool that can decompose a complex signal into a physical one, detaching it from its background while retaining its main features. Its main advantages are simple calculation and fast parallel computing. It includes a Boolean operation, and addition and subtraction without multiplication, and offers easy implementation using hardware. Therefore, this method has been widely used in recent years in the field of signal processing and fault diagnosis. Zhang et al. [17] proposed a multi-resolution morphological gradient method to efficiently extract fault-generated transients and accurately identify fault locations in a power transmission line system. Zhang et al. [18] proposed a multiscale morphology analysis method to extract impulsive features from signals with strong background noise. Luo et al. [19] proposed a new method based on chirplet path pursuit and multiscale morphology analysis for gear fault detection under time-varying rotating speed. Li et al. [20] proposed a continuous-scale mathematical morphology scheme by interpolation and re-sampling to improve scale resolution for more reliable fault signature extraction. Chen et al. [21] proposed a pattern spectrum, obtained from multiscale mathematical morphology, as a feature extraction index. Wang et al. [22] proposed a multiscale morphology analysis method of acoustic emission signal and quantitative diagnosis for bearing fault. Gong et al. [23] proposed an optimized multiscale morphology method based on conventional multiscale morphology and iterative morphology to effectively suppress noise and extract the impulsive features found in the vibration signals of faulty rolling element bearings. Li et al. [24] proposed a novel signal processing scheme, bandwidth empirical mode decomposition, and adaptive multiscale morphological analysis for early fault diagnosis of rolling bearings. Deng et al. [25] proposed a novel method called adaptive multiscale AVG-Hat morphology filter to detect and extract the fault features hidden in the heavy noise of the vibration signal. Xu et al. [26] proposed a fault diagnosis method for rotating machinery based on local mean decomposition morphological filtering and using a least square support vector machine. Liu et al. [27] proposed a performance degradation feature extraction method based on mathematical morphological gradient spectrum entropy. Qu et al. [28] investigated the influence of deformation-band damage zone on reservoir performance in the presence of different fault core transmissibility multipliers. Chen et al. [29] proposed a novel model—deep inception nets with atrous convolution—to extract common features shared by both kinds of data, because the differences between the artificial one and the natural one baffle the learning machine. Li et al. [30] proposed a three-dimensional lumped-parameter nonlinear dynamic model for compound planetary gear set, which takes into consideration time-varying mesh stiffness (TVMS), mesh phase relations, and gear chipping defects. Other fault damage degree identification methods have also been proposed to realize quantitative fault diagnosis [31,32,33,34,35,36,37,38,39,40,41], which offer better results; however, drawbacks, such as lower identification accuracy, longer identification time, and multiple faults with different fault severity degrees, remain.

Entropy is a concept in the thermodynamics field. Shannon introduced the concept to the information field and proposed information entropy in 1948, used to measure uncertainty [42]. This gradually generalized the concept. In recent years, researchers proposed a performance degradation feature extraction method using the gradient spectrum entropy of mathematical morphology based on mathematical morphology, information entropy and fractal theory. The proposed method was applied to the quantitative diagnosis of rolling bearing.

In this paper, on the basis of analyses of mathematical morphology and information entropy, a high-order differential mathematical morphology gradient spectrum entropy is proposed to quantitatively identify the fault damage degree of rolling bearing. Firstly, the vibration data of motor bearings with different fault degrees are collected. Then, according to the change of the mathematical morphology gradient spectrum and the mathematical morphology gradient spectrum entropy, the optimal scale range of the structural element is determined. In order to quantitatively identify the fault damage degree, a high-order differential mathematical morphological gradient spectrum entropy is defined. Discrimination of the fault damage degree is proposed to compare the effectiveness of fault damage degree identification with general mathematical morphological spectrum entropy.

The remainder of the paper is organized as follows. Basic methods are introduced in Section 2. The high-order differential mathematical morphological gradient spectrum entropy is defined in Section 3. In Section 4, a novel fault damage degree identification method, based on high-order differential mathematical morphological gradient spectrum entropy, is proposed. In Section 5, data sources and the experimental environment is introduced. The influence of damage degree identification of the bearing fault under a changing scale is studied and analyzed in Section 6. In Section 7, an application for the motor bearing fault is introduced in detail. Finally, conclusions are offered in Section 8.

## 2. Basic Methods

### 2.1. Mathematical Morphology

Mathematical morphology (MM) is a nonlinear signal processing and analysis tool based on integral geometry and random set theory, proposed by Matheron and Serra [43]. The MM transformation obtains the geometric information of each signal and the relationship between them by using the structure element to extract signal features. The analyzed signals are usually unary functions in the time domain.

The structural element of MM is a real number sequence. According to their shape, structure elements can be categorized as elliptical, triangular, and flat. The basic operations of MM include erosion, dilation, opening and closing. The definitions of these operations are described below. Presume that the original signal *f*(*n*) is defined as a discrete function for *F* = (0, 1, …, *N* − 1), structure elements *g*(*m*) is defined as a discrete function for *G* = (0, 1, …, *M* − 1), and *N* ≥ *M*. Then, the erosion of *f*(*n*) by *g*(*m*) is defined as follows:

(1) (f⊖g)(n)=min[f(n+m)+g(m)]

The dilation of *f*(*n*) by *g*(*m*) is defined as:

(2) (f⊕g)(n)=max[f(n−m)+g(m)]

The opening operation of *f*(*n*) by *g*(*m*) is defined as:

(3) (f∘g)(n)=(f⊖g)⊕g

The closing operation of *f*(*n*) by *g*(*m*) is defined as:

(4) (f•g)(n)=(f⊕g)⊖g

The opening operation is a compound operation of corrosion and then expansion; its effect is to filter the foreground noise in the signal. The closing operation is a compound operation of expansion and then corrosion; its effect is to filter the background noise in the signal.

### 2.2. MultiScale Mathematical Morphology

A multiscale operation uses structure elements of different scales to process and analyze signals [43]. Presume that the original signal *f*(*n*) is defined as a discrete function for *F* = (0, 1, …, *N* − 1), structural elements *g*(*m*) is defined as a discrete function for *G* = (0, 1, …, *M* − 1), and λ is the analytical scale. Then, the structure elements under λ scales are defined as follows:

(5) λg=g⊕g…⊕g

On this basis, the erosion, dilation, opening and closing operations of the multiscale MM for discrete signal sequence *f*(*n*) are defined as follows:

(6) (fΘg)λ(n)=(fΘλg)(n)

(7) (f⊕g)λ(n)=(f⊕λg)(n)

(8) (f∘g)λ(n)=(f∘λg)(n)

(9) (f•g)λ(n)=(f⊕λg)Θg

### 2.3. Mathematical Morphology Gradient Spectrum

Just as frequency spectrum can directly reflect the frequency components in a signal, the morphological spectrum based on multiscale morphological theory can also reflect the shape information of structural elements of different scales in a morphological operation [44].

Presume that *f*(*n*) is a time domain function, and g(m) is a convex structure function. The mathematical morphology spectrum of f(n) is defined as follows:

(10) $$PS(f,\lambda ,g)=\left\{ \begin{array}{ll} -\frac{dA(f\circ \lambda g)}{d\lambda } & \;\lambda \geq 0\\ -\frac{dA(f\circ (-\lambda )g)}{d\lambda } & \;\lambda <0 \end{array}\right.$$

where λg=g⊕⋯⊕g:

(11) A=∑f(n)

Here, the size of a one-dimension discrete signal only takes a continuous integer value.

(12) $$PS(f,\lambda ,g)=\left\{ \begin{array}{ll} dA(f\circ \lambda g)\;-dA(f\circ (\lambda +1)g) & \;\lambda \geq 0\\ dA(f•\lambda g)\;-dA(f•(\lambda +1)g) & \;\lambda <0 \end{array}\right.$$

When λ≥0, the mathematical morphological spectrum refers to its opening operation.

The morphological gradient operator is defined as the difference of using the expansion operation and the corrosion operation between the signal f and the structural element g.

(13) Grad(f,g)=f⊕g−f Θ g

The morphological gradient operator is combined with the mathematical morphology spectrum to obtain a mathematical morphology gradient spectrum, which is defined as follows:

(14) PGS(f,λ,g)=A[Grad(f,(λ+1)g)−Grad(f,λg)] λ≥0

### 2.4. Mathematicall Morphology Gradient Spectrum Entropy (MMGSE)

The morphological spectrum entropy describes the sparsity degree of morphological spectrum values, that is, the order of degree of shape probability distribution of the signal under different morphologies. On the basis of analysis of the probability feature of energy distribution, entropy is applied to the mathematical morphology spectrum to propose a mathematical morphology spectrum entropy (MMSE).

(15) $$PSE\left(f,\lambda ,g\right)=-\overset{\lambda_{\max}}{\underset{\lambda =\lambda_{\min}}{\sum }}q\left(\lambda \right)\ln q\left(\lambda \right)$$

where q(λ)=PS(f,λ,g)/∑PS(f,λ,g), *PS* represents a mathematical morphological spectrum. PSE divided $\ln\left(\lambda_{\max}-\lambda_{\min}+1\right)$ to normalize the mathematical morphology spectrum entropy on the value range [0, 1]. In this paper, the mathematical morphology spectrum entropy is normalized.

The morphological gradient spectrum entropy reflects the morphological features and component changes of signals. It is also a complexity index that describes signals in essence. The mathematical morphology gradient spectrum entropy (MMGSE) is defined as follows:

(16) $$PGSE\left(f/g\right)=-\overset{\lambda_{\max}}{\underset{\lambda =\lambda_{\min}}{\sum }}q\left(\lambda \right)\ln q\left(\lambda \right)$$

where q(λ)=PGS(f,λ,g)/∑PGS(f,λ,g). *PGS* represents a mathematical morphological gradient spectrum entropy. *PGSE* divided $\ln\left(\lambda_{\max}-\lambda_{\min}+1\right)$ to normalize the MMGSE on the value range [0, 1].

## 3. High-Order Differential Mathematical Morphological Gradient Spectrum Entropy

In this paper, a definition of high-order differential mathematical morphological gradient spectrum based on mathematical morphology gradient spectrum and high-order difference is proposed.

(17) G_PGS(f,λ,g,n)=A[Grad(f,(λ+n)g)−Grad(f,λg)]

Grad is the gradient operation of mathematical morphology.

The proof of process is described as follows:

(18) $$\begin{array}{l} PGS(f,\lambda ,g,n)=A[Grad(f,(\lambda +n)g)-Grad(f,(\lambda +n-1)g)]+\cdots \\ +A[Grad(f,(\lambda +2)g)-Grad(f,(\lambda +1)g)]\\ +A[Grad(f,(\lambda +1)g)-Grad(f,(\lambda g)]\\ =\sum [Grad(f,(\lambda +n)g)-Grad(f,(\lambda +n-1)g)]+\cdots \\ +\sum [Grad(f,(\lambda +2)g)-Grad(f,(\lambda +1)g)]\\ +\sum [Grad(f,(\lambda +1)g)-Grad(f,\lambda g)]\\ =\sum Grad(f,(\lambda +n)g)-\sum Grad(f,(\lambda +n-1)g)+\cdots \\ +\sum Grad(f,(\lambda +2)g)-\sum Grad(f,(\lambda +1)g)\\ +\sum Grad(f,(\lambda +1)g)-\sum Grad(f,\lambda g)\\ =\sum Grad(f,(\lambda +n)g)-\sum Grad(f,\lambda g)\\ =A[Grad(f,(\lambda +n)g)-Grad(f,(\lambda +g)] \end{array}$$

The high-order difference mathematical morphological gradient spectrum is equal to the equal interval sampling of the gradient spectrum. The Grad operation is expanded to the mathematical morphology operations of expansion and corrosion to obtain the high-order difference mathematical morphology spectrum. The high-order difference mathematical morphology gradient spectrum entropy based on high-order difference mathematical morphology spectrum and entropy (HDMMGSE) is defined as follows:

(19) $$G\_PGSE(f,\lambda ,g,n)=-\sum_{i=1}^{k*n}q(\lambda )\ln q(\lambda )$$

where q(λ)=PGS(f,λ,g)/∑PGS(f,λ,g). *G*_*PGE* represents a mathematical morphological gradient spectrum entropy. *G*_*PGSE* divided $\ln\left(\lambda_{\max}-\lambda_{\min}+1\right)$ to normalize the HDMMGSE on the value range [0, 1].

## 4. A Novel Fault Damage Degree Identification Method

A variety of fault diagnosis methods have currently been effectively proposed to detect fault types and damage degrees of motor bearing in order to keep machinery performing at its best and avoid abnormal event progression. These qualitative methods mainly use appropriate signal analysis methods to obtain fault damage degree identification methods, such as finite element model method, least square method, modal expansion method, harmonic theory, information entropy and support vector machines. However, these qualitative methods have drawbacks, which include lower identification accuracy, longer identification time, and multiple faults with different fault severity degree. Thus, this paper uses mathematical morphology, multiscale operations and morphological spectrum entropy to propose a damage degree identification method based on high-order difference mathematical morphology gradient spectrum entropy (HMGSEDI). First, the vibration data of motor bearing with different fault severity are collected. Analysis of the features of gradient spectrum enables discussion of the mathematical morphology gradient spectrum and mathematical morphology gradient spectrum entropy under different scales, in order to determine the optimal scale range of structural elements. Next, differentiation degree is defined. The high-order difference mathematical morphological spectrum entropy is calculated under different fault severity. The comparison and analysis of the mathematics morphological spectrum entropy and the high-order difference mathematical morphological spectrum entropy are discussed in detail. This effective quantitative identification method for fault damage degree can offer a novel way to identify fault damage degree and fault prediction of rotating machinery.

**Figure 1:**
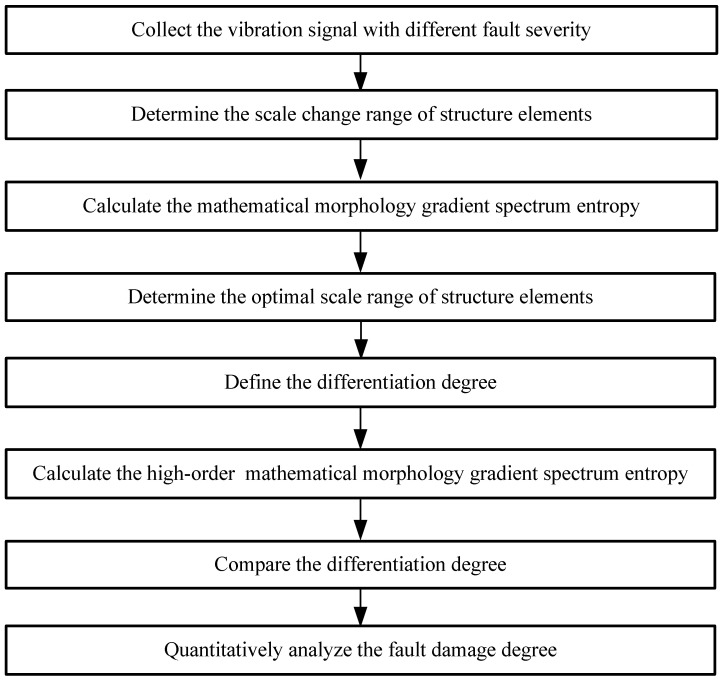
The flow of the fault damage degree identification method.

The flow of the fault damage degree identification method, based on HDMMGSE, is shown in Figure 1.

## 5. Data Sources and Experimental Environment

**Figure 2:**
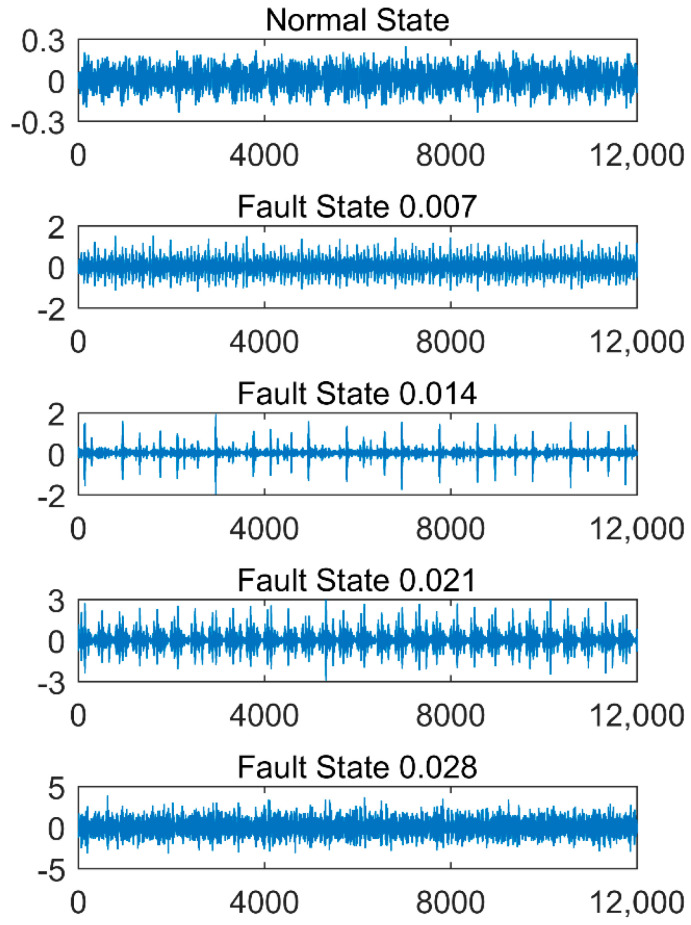
The raw vibration data of five states for motors with no-load.

In order to validate the effectiveness of the proposed method, the authors used vibration data from the Bearing Data Center of Case Western Reserve University [45]. The 6205-2RS 6 JEM SKF deep groove ball bearing was employed for the experiment. A 1.5 kW motor was connected to a dynamometer and torque sensor by self-aligning. Vibration data was collected using accelerometers, which were attached to the housing with magnetic bases. Accelerometers were placed at the 12 o’clock position at both the drive end and fan end of the motor housing. Faults were introduced into the bearing’s inner race using the electro-discharge machining method. The fault diameters were 0.007 inch, 0.014 inch, 0.021 inch and 0.028 inch (1in = 2.54 cm). The vibration data was measured under no-load(0HP) and load(3HP) at rotation speeds (1797 r/min and 1730 r/min). The bearing’s vibration data was sampled at a frequency of 12,000 Hz, and the duration of each vibration signal was 10 s. The points from 12,001 to 72,000 were analyzed under four fault states and a normal state. Each state had 60,000 points, divided into five sets of 12,000 points each. There were 25 sets for four fault states and a normal state. Due to limited space, only the raw vibration data of five states for motor with no-load is presented in Figure 2.

The first dataset of each state is used to obtain the clear gradient spectrum: points from 12,001 to 24,000 are used in this paper. Twenty-five datasets are used to obtain the gradient spectrum entropy. The unit structural element [0 0 0] was used here. The experiment environment is described as Intel Core I5 7300HQ, DDR4 2.4 GHz and 8 GB RAM, Win 7 and Matlab 2014a.

## 6. Analysis of the Influence of Damage Degree Identification of Bearing Fault under a Changing Scale

In this section, the influence of structural elements on fault damage degree identification is discussed in detail. According to changes in the mathematical morphology gradient spectrum and the mathematical morphology gradient spectrum entropy, the optimal scale range of the structural elements is determined. The vibration signals of motors with no-load state and load state are analyzed, respectively.

### 6.1. Analysis of Vibration Signals of Motors with No-Load

The vibration signals of five different states under no-load are analyzed in detail. The unit structural element is [0 0 0]. The scale range of the structural element is 1 to 50.

**Figure 3:**
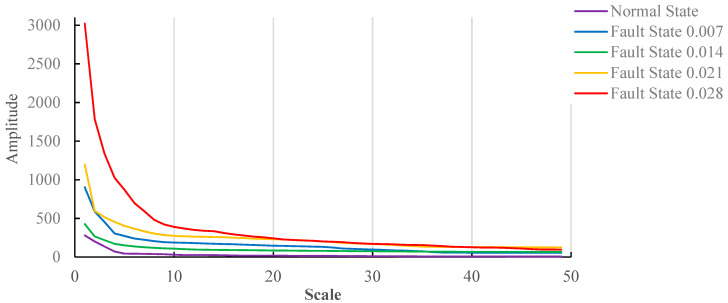
The mathematical morphological gradient spectrum under no-load.

The mathematical morphological gradient spectrum is obtained according to Formula (14); the results are shown in Figure 3.

As can be seen in Figure 3, the morphological gradient spectrum curves under different fault states are clearly distinguished on the 1 to 20 scale. If the fault damage degree is large, the gradient spectrum amplitude is also large. The amplitude difference of the morphological gradient spectrum under different fault states is less, and aliasing occurs to a certain extent on the 20 to 50 scale.

The values of the mathematical morphological gradient spectrum entropy are calculated according to Formula (16). In order to determine the optimal scale range for fault damage degree identification, the minimum structural element scale 2 is kept unchanged, while the maximum element scale is altered. The determination of the maximum scale is mainly based on the gradient spectrum feature, which is reflected as follows:

(1) There is a non-negative lower bound in a whole monotone decreasing sequence.
(2) The front value of the gradient spectrum varies greatly, and the back value of the gradient spectrum varies a little (up to zero).
(3) The gradient spectrum is discrete.

**Figure 4:**
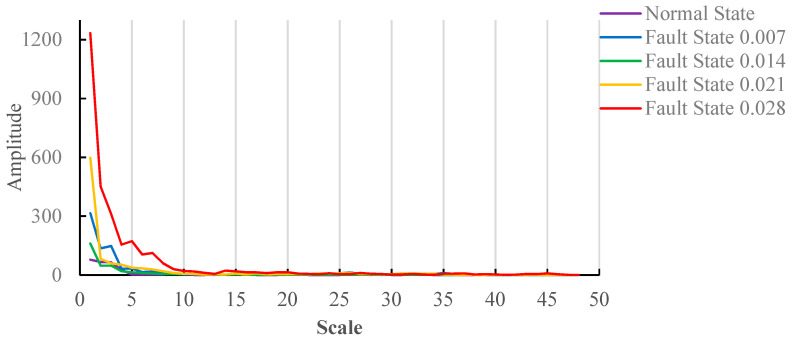
Trend of gradient spectrum under no-load condition.

The larger variation points in gradient spectrum are called feature points. All spectrum lines should contain feature points. *y*(*k +* 1) *− y*(*k*) is used to characterize the variation of the discrete function. Because the gradient spectrum is a decreasing function, *y*(*k*) − *y*(*k* + 1) is used; it offers convenient observation and description. The results are shown in Figure 4.

Figure 4 shows the variation trend of the gradient spectrum under a no-load state. The first point is the gradient spectrum difference between the first point and the second point in Figure 3. Because the curve change of the fault damage degree 0.028 is most obvious, it determines the scale range. As can be seen from Figure 4, the gradient spectrum tends to be stable after the scale value reaches 17. The structural element scale range between 2 and 17, which is a moderate scale range, contains all gradient spectrum feature points. The change of gradient spectrum is less after the scale reaches 17. The structural element scale 50 is selected as an excessive scale range.

**Figure 5:**
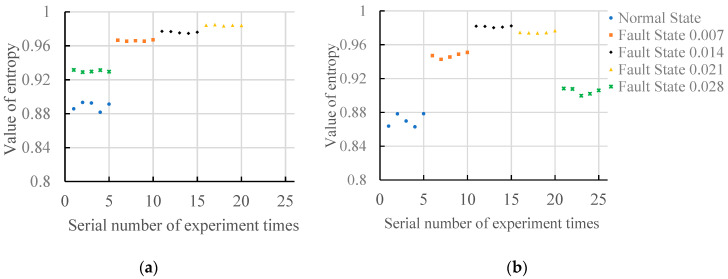
The gradient spectrum under no-load condition. (**a**) Scale of structure element: 2~17; (**b**) Scale of structure element: 2~50.

Figure 5 shows two results of the gradient spectrum entropy of structural elements between 2 and 17 and between 2 and 50 under a no-load state. As can be seen from Figure 5a, when the maximum structural element scale is 17 (except for the fault damage degree 0.028), with increase of the fault damage degree, the values of the gradient spectrum entropy increases, and all values of the gradient spectrum entropy are more than 0.95. When the fault damage degree is 0.028, all values of the gradient spectrum entropy are less than 0.95. As can be seen from Figure 5b, when the maximum structural element scale is 50, the values of gradient spectrum entropy under normal state, fault damage degree 0.007 and fault damage degree 0.014 increase, while the values of gradient spectrum entropy under fault damage degree 0.014, fault damage degree 0.021 and fault damage degree 0.028 reduce. The entropy values of different fault damage degrees are different and can be clearly distinguished.

As can be seen from Figure 4, the values of gradient spectrum entropy under normal state show that the values of gradient spectrum entropy under different fault states are stable. When the maximum structural element scale is 50, the stability of gradient spectrum entropy values under different fault states become bad.

### 6.2. Analysis of Vibration Signals of Motors with Load(3HP)

The vibration signals of five different states under load(3HP) are analyzed in detail. The unit structural element is [0 0 0]. The scale range of the structural element is 1 to 150.

**Figure 6:**
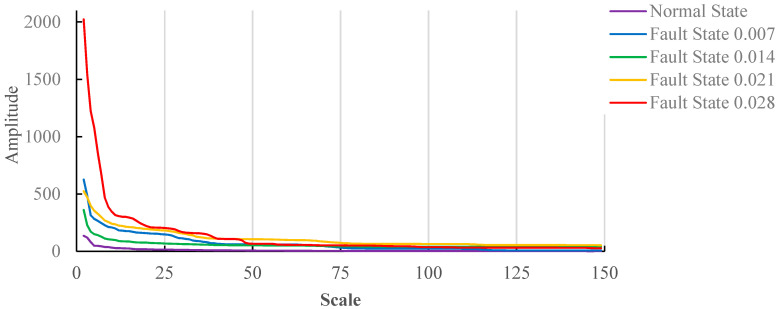
The mathematical morphological gradient spectrum for motors with load(3HP).

The mathematical morphological gradient spectrum is obtained according to Formula (14); the results are shown in Figure 6.

Similar to the no-load state, Figure 6 shows the mathematical morphological gradient spectrum under the load(3HP) state. When the structure element scale is smaller, the mathematical morphological gradient spectrum of fault damage degree 0.021 and 0.007 appears to overlap. The values of the mathematical morphological gradient spectrum entropy are calculated according to Formula (16). In order to determine the optimal scale range for fault damage degree identification, the minimum structure element scale 2 is unchanged, and the maximum element scale is changed.

**Figure 7:**
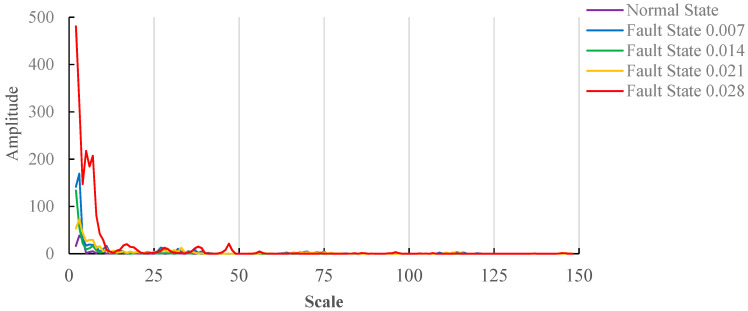
Trend of gradient spectrum under the load(3HP).

Figure 7 shows the variation trend of gradient spectrum under the load(3HP) state, and the first point is the gradient spectrum difference between the first point and second point in Figure 6. Because the curve change of fault damage degree 0.028 is the most obvious, it determines the scale range. As can be seen from Figure 6, the structure element scale 49 shows relatively large changes; therefore, the structure element scale range between 2 and 50, which is a moderate scale range, contains all gradient spectrum feature points. The change of gradient spectrum is less after the structure element scale reaches 50. The structure element scale 150 is selected as an excessive scale range.

**Figure 8:**
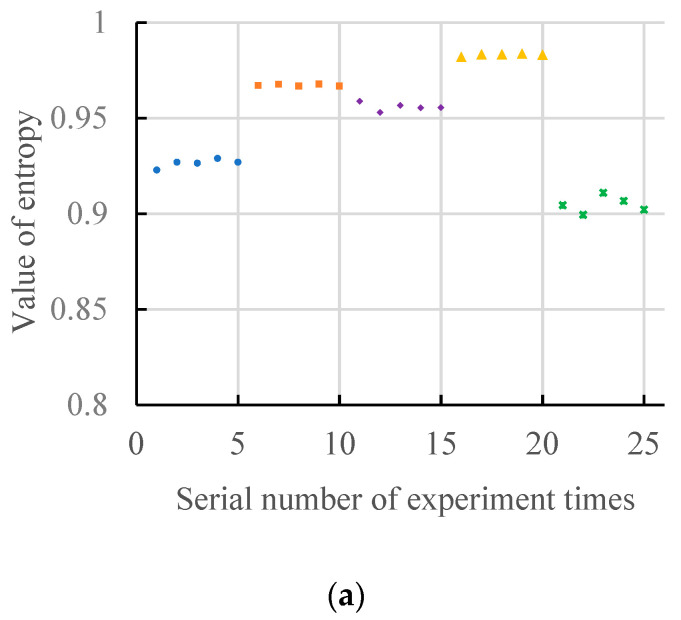

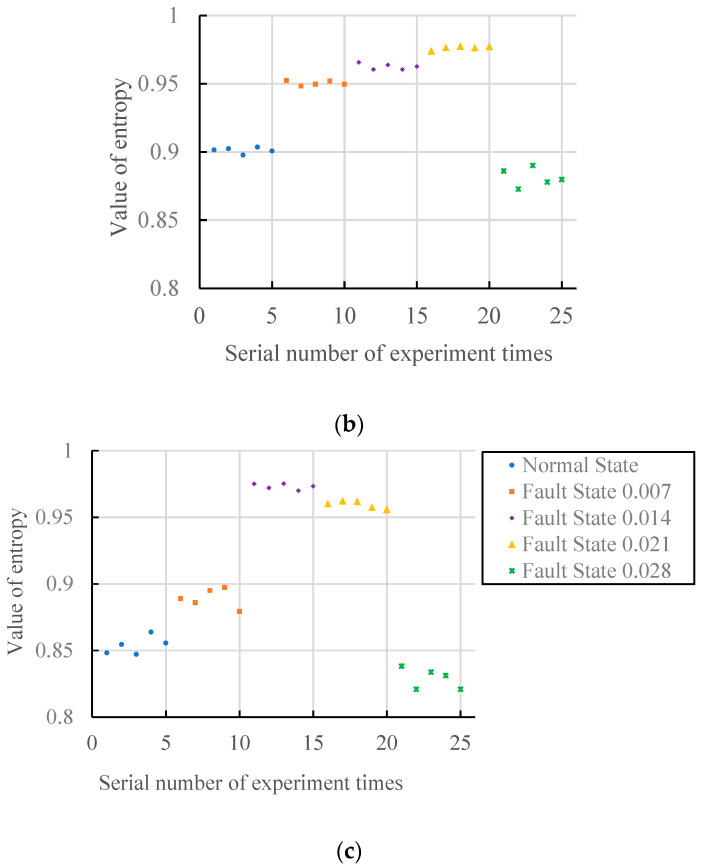
The gradient spectrum under load(3HP). (**a**) Scale of structure element 2~20; (**b**) Scale of structure element 2~50; (**c**) Scale of structure element 2~150.

Figure 8 shows the results of the gradient spectrum entropy of structure elements between 2 and 25, 2 and 50, and 2 and 150 under the load(3HP) state. As can be seen from Figure 8a, when the structure element scale is 20, the values of gradient spectrum entropy under different fault damage degrees are different, and distribution regularity is worse. As can be seen from Figure 8b, when the structure element scale is 50, the values of gradient spectrum entropy under normal state fault damage degree 0.007, 0.014 and 0.021 increase, and the values of gradient spectrum entropy concentrate between 0.94 and 0.98 in the fault states. When the fault damage degree is 0.028, it is similar to the maximum structure element scale 20; the value of gradient spectrum entropy is smaller than that of normal state, and takes on random distribution. As a whole, it shows similar regularity as Figure 5a. As can be seen from Figure 8c, when the maximum structure element scale is 150, the values of gradient spectrum entropy under the normal state fault damage degree 0.007 and 0.014 increase, and the values of gradient spectrum entropy under fault damage degree 0.021 and 0.028 decrease. When the fault damage degree is 0.028, the value of gradient spectrum entropy is smaller than that of the normal state.

As can be seen from Figure 8, when the maximum structure element scale is 50, the values of gradient spectrum entropy under the normal state fault damage degree 0.007, 0.014, 0.021 and 0.028 have better stability and regularity.

### 6.3. Comprehensive Analysis of Experimental Results

The optimal structure element range for motors with no-load and load(3HP) states is different when comparing and analyzing the mathematical morphology gradient spectrum. As can be seen from the experimental results, the motor with load(3HP) state reduces the change rate of the mathematical morphology gradient spectrum. In Figure 3, the curves of different fault damage degrees gently descend after the coordinate point arrives at 25. When the motor operates under load(3HP), it is clearly observed that the curve of fault damage degree 0.028 takes a sudden decline at points of 27, 37 and 47. The curves of other fault damage degrees are much smaller than the curve of fault damage degree 0.028. Therefore, it is not easy to observe other fault damage degrees.

The suitable scale range of structure element is from 2 to 17 for motors with no-load state. The suitable scale range of structure element for motor with load(3HP) state is from 2 to 50. If the scale range of structure elements is too small, the fluctuation of gradient spectrum entropy value under the same fault damage degree is also smaller and relatively stable. However, the distribution of the gradient spectrum is rather disordered in the process of increasing fault damage degree. If the scale range of structure elements is too large, the fluctuation of gradient spectrum entropy value under the same fault damage degree is also larger. However, the distribution of gradient spectrum is relatively neat in the process of increasing fault damage degree. It is helpful to determine the fault damage degree. If the scale range of structure element is suitable, the fluctuation of gradient spectrum entropy value under the same fault damage degree is suitable too. The distribution of gradient spectrum is relatively neat in the process of increasing fault damage degree. For practicality, the best scale range of structure elements should be selected according to actual applications. In the experiment, when the scale range of structure element is larger, the values of gradient spectrum entropy of mathematical morphology show lower computational efficiency, especially for motors with load state.

## 7. Application Case Analysis

Because the mathematical morphology spectrum entropy can quantitatively describe the fault damage degree, the high-order differential mathematical morphology entropy is applied to identify the fault damage degree of motor bearing in this section. In order to quantitatively describe the influence of high-order differential mathematical morphology spectrum entropy on motor bearing fault damage degree, the definition of discrimination is given and described in detail.

First, the mean value of high-order differential mathematical morphology spectrum entropy under one state is calculated using the following expression:

(20) $$G\_PGSE_{mean}=\frac{1}{m}\sum_{i=1}^{m}G\_PGSE_{i}$$

where, *m* is the number of data sets under one state.

Next, the difference between two adjacent states is defined as the discrimination:

(21) $$\Delta_{G}=G\_PGSE_{mean(i+1)}-G\_PGSE_{mean(i)}$$

where, *i* is the status number of equipment.

Similarly, when the mathematical morphological spectrum entropy is used, the definition of discrimination is given and described. The mean value of mathematical morphological spectrum entropy under one state is calculated as:

(22) $$PGSE_{mean}=\frac{1}{m}\sum_{i=1}^{m}PGSE_{i}$$

where, *m* is the number of data sets under one state:

(23) $$\Delta =PGSE_{mean(i+1)}-PGSE_{mean(i)}$$

where, *i* is the equipment status number.

Because the mathematical morphology spectrum entropy has lower calculation efficiency, the high-order differential mathematical morphology spectrum entropy can solve the problem to a certain extent. According to Formula (19), when the high-order difference is used, only the mathematical morphological operations under the structure element scale λ+n and λ are executed, while mathematical morphological operations under other structure element scales are not. This will greatly reduce the amount of calculation.

### 7.1. Analysis of Motors with No-Load State

**Figure 9:**
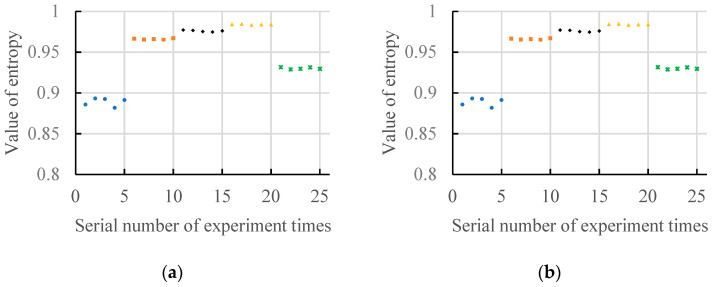
The gradient spectrum in the structure element scale range between 2 and 17 under no-load state. (**a**) Serial number of experiment times *n* = 1. (**b**) Serial number of experiment times *n* = 5.

The comparison between the general gradient spectrum entropy and the high-order differential spectrum entropy in the structure element scale range between 2 and 17 under no-load state is shown in Figure 9.

Figure 8 shows a comparison between the general gradient spectrum entropy (*n* = 1) and the high-order differential spectrum entropy (*n* = 5) in the structure element scale range between 2 and 17 under no-load state. For convenient comparison, Figure 9a,b use the same coordinate system, and the same fault type uses the same legend. As can be seen from Figure 9a, the distribution of the general gradient spectrum entropy value and the high-order differential spectrum entropy value are similar, and the values are slightly different. The quantitative analysis results of the discriminations of two gradient spectrum entropy is shown in Table 1.

As can be seen from Table 1, the discrimination of high-order differential gradient spectrum entropy is larger than the discrimination of general gradient spectrum entropy under no-load state. The calculation time of the general gradient spectrum entropy is 21.205 s (Figure 8a). The calculation time of high-order differential gradient spectrum entropy is 5.341 s in (Figure 8b). The results show that calculation efficiency is significantly improved by using the high-order differential gradient spectrum entropy.

### 7.2. Analysis of Motors with the Load(3HP) State

**Figure 10:**
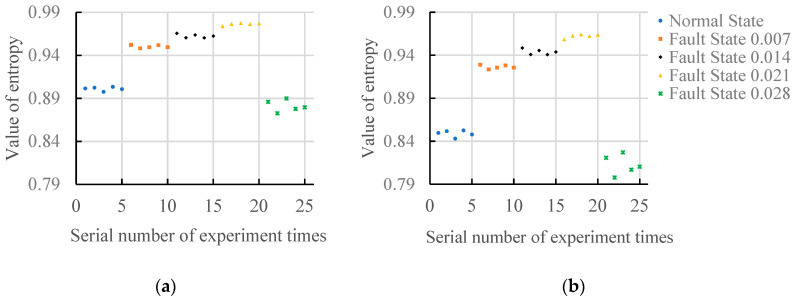
The gradient spectrum using the structure element scale range between 2 and 50 under load(3HP). (**a**) Serial number of experiment times *n* = 1. (**b**) Serial number of experiment times *n* = 5.

A comparison between the general gradient spectrum entropy and the high-order differential spectrum entropy in the structure element scale range between 2 and 50 under load(3HP) state is shown in Figure 10.

Figure 10 shows the comparative results of the general gradient spectrum entropy (*n* = 1) and the high-order differential spectrum entropy (*n* = 5) in the structure element scale range between 2 and 50 under load(3HP) state. For convenient comparison, Figure 10a,b use the same coordinate system, and the same fault type uses the same legend. As can be seen from Figure 10, the discriminations of two gradient spectrum entropy are different. The quantitative analysis results of the discriminations of two gradient spectrum entropy under load state is shown in Table 2.

As can be seen from Table 2, the discrimination of high-order differential gradient spectrum entropy is larger than the discrimination of general gradient spectrum entropy under the load(3HP) state. The calculation time of general gradient spectrum entropy is 66.597 s (Figure 10a). The calculation time of high-order differential gradient spectrum entropy is 9.468 s (Figure 10b). The experimental results show that calculation efficiency is significantly improved by using the high-order differential gradient spectrum entropy.

### 7.3. Comprehensive Analysis of Experimental Results

The influence of the high-order differential mathematical morphology spectrum entropy on bearing fault damage degree identification is studied. Discrimination is used to quantitatively describe the difference between the fault damage degrees. As can be seen from Table 1 and Table 2, the high-order differential morphological spectrum entropy can increase the discrimination of entropy values under different fault damage degrees. For example, when the morphological gradient spectrum entropy is used to calculate the gradient spectrum entropy values in the scale range of structure element between 2 and 17 under no-load state, the gradient spectrum entropy values of fault damage degrees 0.007, 0.014 and 0.021 are concentrated between 0.94 and 0.97. These entropy values are very close to maximum value 1. It is not detrimental to effectively recognize fault damage degree. After difference order *n* is improved, the gradient spectrum entropy values between different fault damage degrees increase to profitably recognize the fault damage degree. The high-order differential mathematical morphology spectrum entropy significantly increases calculation efficiency. It is roughly proportional to the improved operation efficiency and the difference order *n*.

## 8. Conclusions

(1) On the basis of analysis of the mathematical morphology spectrum and high-order difference, the definition of high-order differential mathematical morphological spectrum is proposed in this paper. The entropy technique is introduced into the high-order differential mathematical morphological spectrum to propose the definition of high-order differential mathematical morphological spectrum entropy.

(2) The defined high-order differential mathematical morphological spectrum entropy is used to discuss and analyze the scale change influence of structure element in fault damage degree identification for motors with no-load and load states. According to the varied characteristics of the mathematical morphology gradient entropy, the appropriate scale range of the structure elements in fault damage degree identification is determined. The results show that the optimal scale range of structure elements is different for effective identification of fault damage degree under different load conditions. For practicality, the optimal scale range of structure elements should be analyzed in actual applications.

(3) The fault damage degree identification, based on high-order differential mathematical morphological spectrum entropy, has been studied in depth. The discrimination of fault damage degree has been defined to quantitatively describe the difference in fault damage degree identification between the high-order differential mathematical morphological spectrum entropy and general mathematical morphological spectrum entropy. The analysis shows that high-order differential mathematical morphology entropy can effectively identify fault damage degree and greatly improve operational efficiency.

Therefore, the proposed method is novel and effective quantitative identification method that helps identify fault damage degree and predict fault in rotating machinery.

## Figures and Tables

**Table 1:** Comparison of the discriminations of two gradient spectrum entropy under no-load state.

Fault Damage Degree	Mean Value		Discrimination	
	$G\_PGSE_{mean}\;(n=5)$	$PGSE_{mean}\;(n=1)$	$\Delta_{G}\;(n=5)$	Δ (n=1)
Normal state	0.8352	0.8889	/	/
Fault damage degree 0.007	0.9442	0.9660	0.1090	0.0771
Fault damage degree 0.014	0.9552	0.9759	0.0110	0.0099
Fault damage degree 0.021	0.9677	0.9840	0.0125	0.0081
Fault damage degree 0.028	0.8626	0.9302	−0.1051	−0.0538

**Table 2:** Comparison of the discriminations of two gradient spectrum entropy under load(3HP).

Fault Damage Degree	Mean Value		Discrimination	
	$G\_PGSE_{mean}\;(n=5)$	$PGSE_{mean}\;(n=1)$	$\Delta_{G}\;(n=5)$	Δ (n=1)
Normal state	0.8221	0.9011	/	/
Fault damage degree 0.007	0.9100	0.9503	0.0879	0.0492
Fault damage degree 0.014	0.9387	0.9626	0.0287	0.0123
Fault damage degree 0.021	0.9543	0.9763	0.0156	0.0137
Fault damage degree 0.028	0.7831	0.8813	−0.1712	−0.0950
